# Orally Administered Zinc Gluconate Induces Tight Junctional Remodeling and Reduces Passive Transmucosal Permeability Across Human Intestine in a Patient-Based Study

**DOI:** 10.3390/ijms26178540

**Published:** 2025-09-02

**Authors:** Elizabeth A. Del Rio, Mary Carmen Valenzano, Katherine M. DiGuilio, Elizabeth Rybakovsky, Stephanie Kjelstrom, Georgia Montone, Giancarlo Mercogliano, Gary Newman, Patricia Wong, Nicole Albert, Victoria Burris, Kelly Szymanski, Amanda Rodriguez, Erin Hollis, Andrew Kossenkov, James M. Mullin

**Affiliations:** 1 Lankenau Institute for Medical Research, 100 Lancaster Avenue, Wynnewood, PA 19096, USA; edelrio863@gmail.com (E.A.D.R.); valenzanomarycarmen@gmail.com (M.C.V.); kdiguilio@gmail.com (K.M.D.); volleyliz36@gmail.com (E.R.); kjelstroms@mlhs.org (S.K.); georgia.montone@gmail.com (G.M.); 2 Division of Gastroenterology, Lankenau Medical Center, Wynnewood, PA 19096, USA; mainlinemerc@msn.com (G.M.); newmang@mlhs.org (G.N.); wongp@mlhs.org (P.W.); albertn@mlhs.org (N.A.); 3 Department of Medicine, Lankenau Medical Center, Wynnewood, PA 19096, USA; victoriamburris@gmail.com (V.B.); kovacikk@mlhs.org (K.S.); hollise@mlhs.org (E.H.); 4 The Wistar Institute of Anatomy and Biology, University of Pennsylvania, Philadelphia, PA 19104, USA; akossenkov@wistar.org

**Keywords:** tight junction, intestine, claudin, micronutrient, leaky gut, zinc, D-lactate

## Abstract

This study focuses on the issue of whether orally administered zinc (gluconate) (26 mg BID) can induce the remodeling of gastrointestinal barrier function and reduce passive leak across the human intestinal mucosal barrier in situ. Increased transmucosal leak has been implicated in diseases as diverse and seemingly unconnected as Inflammatory Bowel Disease (IBD), Celiac Disease, Autism Spectrum Disorders and Alzheimer’s Dementia. Our current investigation represents the first patient-based study to examine the effect of zinc on gastrointestinal epithelial tight junctions and gastrointestinal barrier leak in otherwise healthy test subjects. Using independent test subject groups for each endpoint, three separate molecular analyses indicated that zinc treatment can achieve a positive outcome: (1) RNA-seq analyses of intestinal biopsies showed salutary patterns of gene transcription changes dealing with not only transcripts of junctional proteins but also transcripts mitigating the proinflammatory state, as well as dedifferentiation (both modulators of tight junction permeability); (2) Western immunoblot analyses of intestinal tissue indicated that tight junctional protein expression was being modified by the administered zinc, most notably Claudin-2 and Tricellulin; (3) zinc treatment induced a reduction in serum levels of a functional marker of passive intestinal leak, namely the GI microbiome metabolite D-Lactate. The data collectively suggest that orally administered zinc can induce remodeling of the intestinal epithelial barrier, resulting in the reduction in GI barrier leak. The overall safety and economy of supplement levels of zinc suggest that this micronutrient could be efficacious as an adjuvant therapy to reduce the condition known as leaky gut, and possibly therefore be protective regarding diseases postulated to involve leaky gut.

## 1. Introduction

The importance of epithelial barrier function to human physiology cannot be overstated. Our anatomy shows humans to be a parallel array of sacs and tubes, each defining an organ-specific luminal compartment, separated from the vascular compartment by an epithelial cell layer interface, very often just one cell layer thick. The ability of that interface to serve as a functional barrier separating very different luminal versus abluminal milieus is critical to all higher animal life. The compromise of that barrier function is a lynchpin throughout a very wide array of disease etiology and is at the core of a great deal of disease-related morbidity and mortality. Although not the sole arbiter of epithelial barrier function, the apically situated, circumferential tight junctional (TJ) complex (*Zonula Occludens*), plays a central role as the dynamic, highly regulated molecular sieve that determines size and charge selectivity along the paracellular transepithelial pathway. Dysregulation or outright failure of this key barrier element is a prominent feature in a very wide array of human disease [1,2].

The gastrointestinal epithelial barrier holds two important hallmarks of its own among our other epithelial tissue barriers. Even surpassing the epidermis, it is the largest epithelial barrier in terms of overall surface area. Secondly, it separates the vasculature from the greatest prokaryotic population in our body, the GI microbiome. Given that the vasculature is home to our immune system, this is a barrier separation that is highly pivotal and whose compromise would a priori result in significant inflammation. Disease-related compromise of GI barrier function is obvious in conditions such as Crohn’s Disease and Ulcerative Colitis [3,4]. However, serious GI barrier compromise often begins very subtly with a focused dysregulation of the TJ complex itself. This occurs very early in diseases as disparate as colon neoplasia [5] and certain intestinal microbial infections [6].

The condition or syndrome known as “Leaky Gut” (LG) is not a recognized disease or even a diagnosis. A cursory examination of it in the literature will be sure to include words and phrases such as “hypothetical,” “may,” “difficult,” and “unrecognized.” Although controversy surrounds it, no one seems to dispute that those conditions of increased intestinal permeability are quite real [7,8]. This is certainly not surprising given that pathophysiologically increased permeability or “leak” across renal tubular barriers [9], oral epithelial barriers [10], airway epithelial barriers [11] and retinal epithelial barriers [12] among many, are fully accepted. No one disputes that GI inflammation can induce increased GI permeability [13]. The controversy may lie in whether GI leak is a necessary and sufficient cause for GI inflammation [14]. And the next question to ask is whether GI leak can be playing a role in seemingly highly unrelated (and physically remote) conditions such as cognitive disease [15].

A consensus appears to be building in the published literature that, at the very least, a serious look into the origins of GI barrier leak, its ramifications, and what one might possibly do to alleviate it is fully warranted. This current investigation deals with the third endeavor—are there therapeutic options by which such leak can be reduced? Any epithelial barrier relies on its TJ complexes as the final gatekeeper of transepithelial paracellular movement, but the GI barrier also involves a wide array of components ranging from mucus layers to the rate (and manner) of epithelial cell turnover within the barrier by necrotic/apoptotic/autophagic means [5,16]. When one considers the complexity of the TJ (26+ claudins, Occludin, Tricellulin, 30+ TJ-related proteins) AND the above-mentioned (non TJ) contributors to the barrier as well, it is very difficult for a de novo drug to achieve barrier improvement. Such a drug must decrease TJ permeability but also not adversely affect the other barrier contributors [17]. A structurally highly diverse set of micronutrients has however succeeded in achieving this goal [18,19]. This success may derive in part from the fact that animal life—and its need for barrier function and protection—has coevolved with these micronutrients in the animal diet. Natural selection may well have vetted these specific micronutrients to succeed here. The ability of Quercetin, Calcitriol and Retinoic Acid to improve barrier function in the airway epithelium [20,21], and of zinc (Zn) to improve and protect the GI barrier [22] have been well documented examples in many cell culture and animal model studies.

The success of Zn in specifically GI barrier function studies has, however, not been well validated in clinical, patient-based studies. In fact, there is only one patient-based study reported in the published literature that shows orally administered Zn decreasing barrier leak in humans. Sturniolo et al. (2001) showed that administration of a similar oral dose (25 mg Zn, 3 times daily) to that used in our current study, reduced the GI barrier leak that accompanies Crohn’s Disease [23]. However, this pathophysiological situation obviated addressing whether Zn could improve *normal* GI barrier function and whether such improvement could trace itself to induced remodeling of the TJ complex. Answering those questions becomes highly important in determining if supplemental Zn can serve a prophylactic, preventative clinical role in a variety of diseases that could trace themselves to aberrantly increased GI leak.

The objectives of this patient-based study are as follows: (1) examining duodenal mucosal biopsies, determine if a twice daily dose of 26.6 mg of zinc produces a molecular effect generally on duodenal epithelia in situ; (2) determining by RNA-seq analyses if a specific effect of zinc occurs on duodenal epithelial transcripts involved in tight junctional barrier function; (3) determining by Western immunoblot analyses if an effect of zinc occurs on tight junctional proteins; (4) determining if zinc produces an effect on gastrointestinal barrier function by measuring blood levels of the microbiome metabolite, D-Lactate.

## 2. Results

### 2.1. Demographics and Zinc Administration

There were three separate patient-based studies conducted within this overall project. Each involved treating gastroenterology patients presenting for esophageal or gastric screening endoscopy or treating healthy test subjects with orally administered Zn for 7–14 days followed by collection of either duodenal biopsy tissue samples or blood samples. Each utilized separate, independent groups of patients. These studies had as their endpoints: (1) RNA-sequencing (microarrays); (2) protein Western immunoblots; (3) serum D-Lactate analyses. Each used a separate, unique set of patients/test subjects. In the first group, patients with a history of Barrett’s Esophagus or GERD (but no intestinal disease or abnormality) and scheduled for surveillance endoscopy, were placed on a Zn gluconate (or sodium gluconate [placebo]) regimen for 14 days prior to their procedure (as described in Materials and Methods). Duodenal mucosal biopsies were then obtained from the bulb portion of the duodenum, flash frozen in the Endoscopy Procedures Room then later thawed, RNA extracted and RNA-seq analyses performed. The demographic characteristics of the patients used in this RNA-seq study are shown in Table 1. The demographics of a second, independent group of similar patients whose biopsies were analyzed by Western immunoblot analyses of specific TJ proteins are also shown. Finally, a third independent group, this time of healthy control subjects with no gastrointestinal or autoimmune disease, were also administered Zn gluconate for 7 days prior to having blood withdrawn for analyses of a biomarker of passive GI permeability/leak, namely the D (dextrorotary) form of lactate (D-Lactate).

In all cases, recruitment consisted of males or females of ages 18–80, of any race/ethnicity and with no active or recorded clinical history of intestinal disease. In each study, enrollment excluded persons whose GI pathophysiology could affect the parameters under study. Therefore, persons with prior or current GI disease that could affect intestinal barrier function (e.g., Crohn’s Disease, Ulcerative Colitis, Celiac Disease as well as current or recent GI viral infection or consumption of medications that are known to affect intestinal permeability such as NSAIDS, steroids, etc.) were all excluded. Alcohol use was restricted. Enrollees were also carefully instructed to refrain from foods (e.g., spices) that are known to affect GI barrier function at least 2 days before duodenal biopsy sampling or blood sampling. In addition, enrollees were instructed to refrain during the period of Zn administration from foods known to interfere with Zn uptake, such as foods containing phytates (e.g., whole grains, nuts, seeds), as well as citrus. Likewise, enrollees were told to discontinue any over-the-counter Zn supplements a week before starting the study, and not to take any Zn supplements (such as Zn included with many multi vitamins) other than the study-provided Zn medication.

In the mRNA and Western immunoblot studies, patients were administered Zn gluconate in lozenge form (26.6 mg BID). In the D-Lactate study, test subjects were administered Zn gluconate in tablet form (30 mg BID). The total daily dose of approximately 60 mg was chosen because of its demonstrated efficacy in our earlier patient-based study involving Zn administration to Barrett’s Esophagus patients, and its effect on Barrett’s epithelia [24]. There were no adverse effects observed that would be attributable to the Zn administration in any of the three sub studies reported here.

Age ranges and gender distributions were similar between placebo and Zn-treated groups. The study population was preponderantly Caucasian due to both the catchment area of the medical center and the frequency of Barrett’s Esophagus in the participating (endoscoped) patients.

### 2.2. mRNA Expression

**Figure 1:**
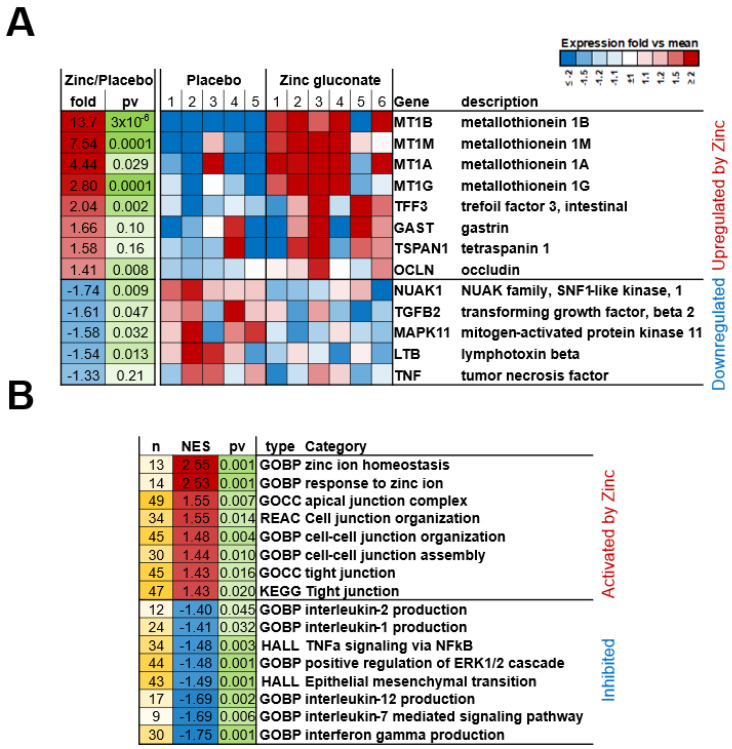
Transcriptional Changes in Duodenal–Epithelial Biopsy Tissue in Response to Zinc-Gluconate Treatment. Six patients (“zinc gluconate”) were treated with orally administered Zn gluconate (26 mg Zn BID) for 14 days prior to upper endoscopy and duodenal mucosa biopsy as described in Materials and Methods. Five patients (“placebo”) were treated with a molar equivalent amount of sodium gluconate BID. RNA was extracted from the biopsy samples and RNA–RNA-seq analyses were performed as described. Gene Set Enrichment Analysis was performed by GSEA as described [25]. (**A**). Expression heatmap of select individual genes related to Zn binding and TJ regulation. Fold; fold increase or decrease relative to transcription level in placebo-treated tissue; pv = DESeq2 *p* value; color intensity represents relative expression levels (vs placebo average) across individual patients: red—relative upregulation; blue—relative downregulation. (**B**). Select categories of genes enriched in response to Zn gluconate treatment as identified by Gene Set Enrichment Analysis (GSEA). These are common functional categories enriched among genes affected by Zn gluconate treatment. Among other Zn-induced patterns unrelated to epithelial barrier function (unreported here; see Supplementary Materials), a pattern of *upregulations* of gene transcriptions relating to epithelial junction integrity was observed in the duodenal tissue samples. A pattern of associated *downregulations* in modifiers of the proinflammatory state, known junctional signaling pathways such as ERK 1/2, and general modifiers of dedifferentiation, was also in evidence. Those downregulations could be consistent with improved junctional barrier function. n = number of leading-edge genes; NES = normalized enrichment score; pv = *p* value of the enrichment. Category types: GOBP = Gene Ontology Biological Process; GOCC = Gene Ontology Cellular Component; REAC = REACTOME pathways; KEGG = KEGG pathways; HALL = HALLMARK gene sets.

RNA was purified from duodenal biopsies, and messenger RNA-seq experiments were performed as described in Materials and Methods. Expression levels of genes in duodenal mucosal biopsy samples from 6 patients treated with Zn gluconate versus 5 patients treated with a sodium gluconate placebo were compared. Our initial focus was whether a pharmacologically effective dose of Zn had been administered, specifically regarding the target tissue—the duodenal epithelia. We observed significant upregulation of the metallothionein genes 1A, 1B, 1G and 1M—with upregulated transcriptions ranging from 2.8 to 13.7-fold (Figure 1A, upregulated genes)—a good indication that the administered Zn dosage was sufficient to affect the targeted epithelial population. There was some patient-to-patient variability in the response to Zn (shown in the individual blocks of the heat map), resulting in varied statistical significance. Of general biological interest was the 2-fold increased transcription of Trefoil Factor 3 and the 1.6-fold increase for gastrin in the Zn-treatment group. Concerning an early indication of a Zn effect on barrier function of the duodenal epithelium, it was noted that the gene transcription of Tetraspanin 1 and Occludin were both increased by approximately 1.5-fold, with the Occludin result having a *p* value = 0.008. Assayed by RNA-seq gene expression, data generated in this study for over 5000 mRNA transcripts is included as an Excel file in Supplementary Material.

Many transcripts were also downregulated in the Zn-treated group. These tended to include indicators and mediators of inflammation (Tumor Necrosis Factor and Lymphotoxin beta) as well as signaling intermediates that are known to be involved in TJ leakiness (Mitogen Activated Protein Kinase, Transforming Growth Factor beta, and the NUAK family (Figure 1A, downregulated genes).

Perhaps the best case for Zn having affected barrier function in this study, as evidenced at the transcriptional level, came from Gene Set Enrichment Analysis (GSEA) of genes affected by Zn treatment. We analyzed the mRNA changes using GSEA software (https://www.gsea-msigdb.org/gsea/index.jsp accessed on 18 August 2025) [25] to find categories that demonstrate significant activation or inhibition patterns based on mRNA expression changes in their member genes in Figure 1B. This analytical method focuses on gene sets or groups that share common biological function or regulation, and showed significant activation in not only the expected categories (Response to Zinc Ion and Zinc Ion Homeostasis), but also indication of the Zn-treatment having induced an effect on duodenal genes associated with the TJ complex (KEGG Tight Junction) or gene ontology cellular components (Tight Junction and Apical Junction Complex), as well as the biological process category, Cell–Cell Junction Assembly, among others. GSEA also revealed noteworthy patterns of inhibition of several categories (Figure 1B) such as biological processes Interleukin 1 and 2 Production, Hallmark [gene set] TNFα Signaling Via NFκB, and the Interferon Gamma Production biological process, among others. The general pattern would indicate downregulation of proinflammatory status, a state that is known to associate with epithelial barrier leak, and in agreement with results seen with specific individual genes in Figure 1A.

### 2.3. Western Immunoblot Analyses

**Figure 2:**
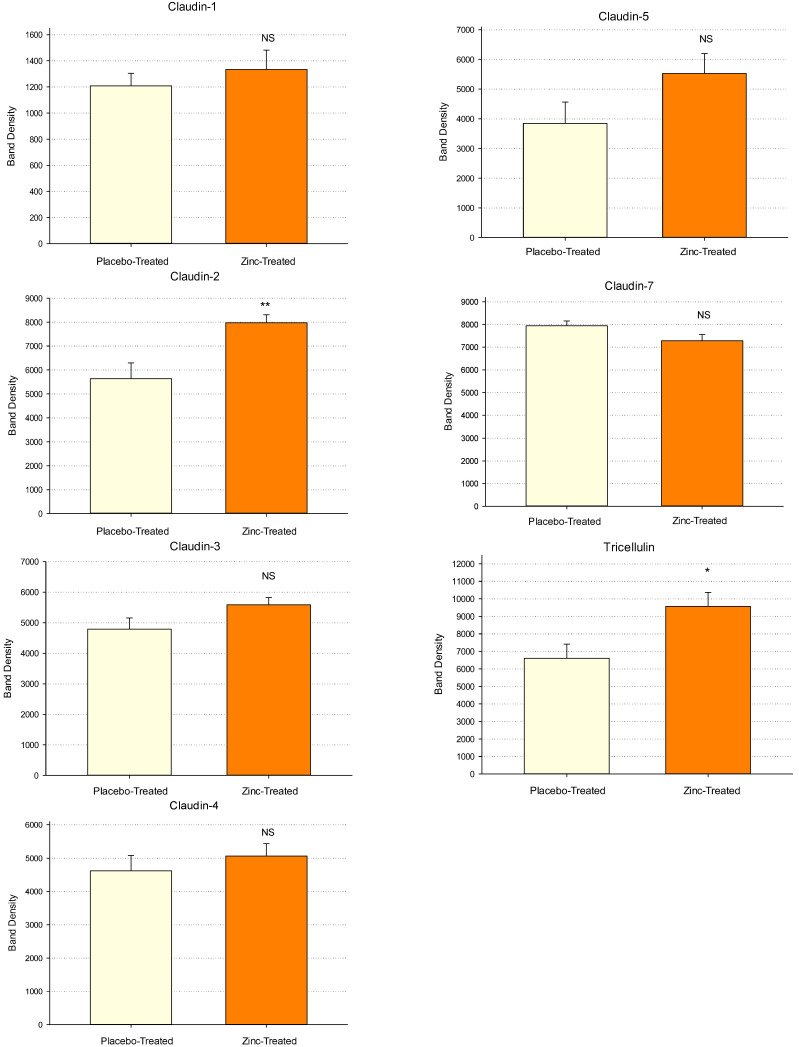
Western Immunoblot Analyses of Tight Junctional Proteins in Duodenal Mucosal Biopsies as a Result of Orally Administered Zinc Treatment. Eleven patients were treated with orally administered Zn gluconate (26 mg Zn BID) for 14 days prior to upper endoscopy and duodenal mucosa biopsy as described in Materials and Methods. Twelve patients were treated with a molar equivalent sodium gluconate placebo. Whole cell lysates of the biopsy tissue were performed as described in Materials and Methods, followed by PAGE and Western immunoblot for seven specific TJ proteins. Densitometry was performed as described to quantify results. All results were normalized to total protein based on MEMCODE assays. Bars represent the mean ± standard error of normalized band densities for 12 patients (placebo-treated) and 11 patients (zinc-treated). Significance was assessed by two-sided Student’s *t*-tests (NS: Not Significant; * *p* < 0.05; ** *p* < 0.01).

Duodenal biopsy samples (from a separate cohort of upper endoscopy patients) were also analyzed at the *translational* level for Zn-induced changes in specific TJ proteins. Duodenal bulb biopsy tissue samples from placebo- and Zn-treated patients were homogenized in lysis buffer, followed by ultracentrifugation, PAGE and immunoblot as described in Materials and Methods. Figure 2 shows the results of densitometry analysis of Western immunoblot protein bands of 7 specific TJ proteins analyzed in biopsy samples from 12 placebo-treated patients and 11 Zn-treated patients. In these bar charts, the mean of the band densities is shown ± the standard error of the mean. Statistically significant increases (*p* = 0.006) were observed for Claudin-2 (40% average increase) and Tricellulin (45% average increase) in the Zn-treatment group (*p* = 0.018). Near significant upregulations (*p* < 0.15) were observed for Claudin-3 (17% average increase) (*p* = 0.086), Claudin-5 (44% average increase) ((*p* = 0.104) and Claudin-7 (10% average decrease) (*p* = 0.072). Claudins-1 and -4 showed no significant change or trend as a result of Zn treatment. Considering the demographic variability existing within and between each treatment group (age, gender, genetics, medications, etc.), along with the relatively small sample size, achieving statistical significance is very challenging in smaller pilot studies such as this current study.

The duodenal biopsy samples used in these immunoblot analyses were taken from a separate set of placebo- and Zn-treated patients than those used in the transcriptional analyses shown in Figure 1A and 1B. It thus becomes noteworthy that the protein data alludes to a similar outcome as the mRNA data, namely TJ complexes remodeled as a result of Zn treatment.

### 2.4. Serum D-Lactate Analyses

**Figure 3:**
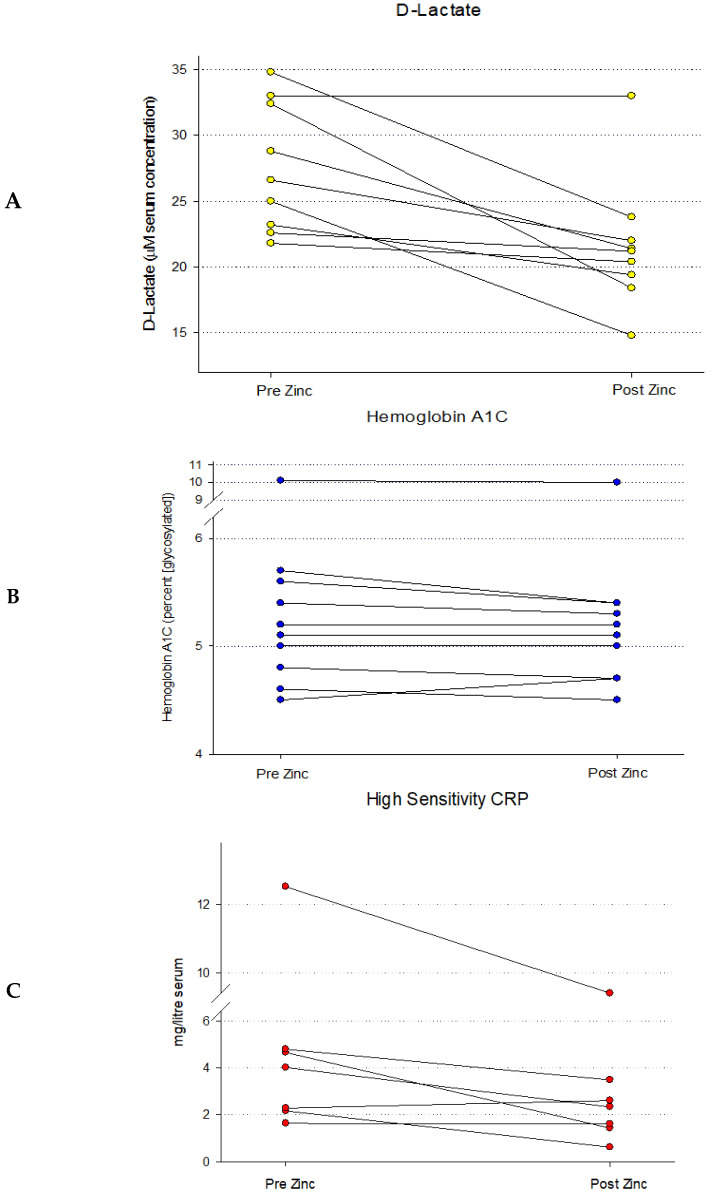
Blood Levels of D-Lactate, Hemoglobin A1C and High-Sensitivity CRP Before and After a 7-Day Regimen of Orally Administered Zinc. Healthy control test subjects (*n* = 9) were treated with Zn gluconate as described in Materials and Methods. Peripheral blood samples were taken before and after the Zn regimen. Results are shown for serum levels of the GI permeability marker D-Lactate (Panel (**A**)), along with two unrelated serum parameters, Hemoglobin A1C (Panel (**B**)) and High Sensitivity CRP (Panel (**C**)), determined spectrophotometrically as described. Whereas no obvious trend was evident for Hemoglobin A1C as a result of Zn treatment, the post-Zn levels of D-Lactate were significantly reduced (*p* = 0.008 [paired Student’s *t*-test]). A statistically significant reduction was also observed for High Sensitivity CRP (*p* = 0.047).

In a third, fully separate patient-based study with a unique complement of test subjects, a 7-day, 30 mg BID Zn treatment was tested for its effect on a *functional* indicator of the GI barrier (Figure 3). This sub-study was undertaken to determine if the Zn-induced changes to structural elements of GI barrier function in Figure 1 and Figure 2 correlated with a change in passive GI permeability (“leak”). A third independent group of healthy control, test subjects (*n* = 20), in this case with no active gastrointestinal disease or history of GI surgery were treated with Zn as described above. Peripheral blood samples were taken before and at the end of the Zn treatments. A placebo group was not used or needed here because of each test subject’s ability to serve as his/her own control in “before” vs. “after” measurements. Serum was collected and analyses of D-Lactate were performed as described, along with analyses of Hemoglobin A1C and High Sensitivity CRP as reference markers that would be unexpected to change with a 7-day Zn treatment. D-Lactate was chosen as a GI permeability biomarker because of its relative ease of analysis and it being a well-documented marker of passive GI permeability [26].

Initial analyses of the entire data set of 20 test subjects showed no discernible trend or statistical significance concerning pre- versus post-Zn treatment. However, when results for those test subjects that showed above average pre-Zn D-lactate leak (those test subjects whose pre-Zn D-lactate levels were above the group median) were observed and analyzed separately, the results shown in Figure 3A were observed. For those 9 test subjects, a clear trend toward lower post-Zn D-Lactate levels could be seen. Using a paired Wilcoxon signed rank test for comparison between pre- and post-Zn, the median D-Lactate decreased significantly from 26.6 (IQR 23.2–32.4) to 21.2 (IQR 19.4–22.0) (*p* = 0.0008). Of those 9 test subjects, only one showed no decrease in their serum D-Lactate level (no change) and this test subject was later found to be pre-diabetic (determined clinically for the first time, with an A1C level over 12). The average decrease in serum D-Lactate concentrations for all 9 test subjects was 21 % (±5% SEM, *n* = 9, *p* = 0.008), post-Zn. The maximally decreased D-Lactate serum level (post-Zn) was 43% and the lowest was 0% (no change). It should be noted that of the original 10 test subjects whose serum D-Lactate levels (pre-Zn) were above the median, one test subject was removed from consideration because a post-study survey revealed that they were taking an iron supplement along with the Zn being taken for this study, and that interference of Zn absorption by the iron was possible [27,28].

In addition to the serum level of the leak biomarker, D-Lactate, the serum components, Hemoglobin A1C and High-Sensitivity CRP (HS CRP) were also measured in the same blood samples. These were chosen because they were viewed to be serum components that were unlikely to be affected by Zn administration. As shown in Figure 3B, levels of Hemoglobin A1C were virtually identical, pre- and post-Zn, showing no change as a result of Zn administration, as anticipated. The results shown here were specifically for the half of test subjects whose initial (pre-Zn) A1C levels were above the median (like the above-median group analyzed for D-Lactate in Figure 3A). Analysis of High Sensitivity CRP exhibited a very different pattern, and was similar to the D-Lactate results. Again, selecting the half of test subjects whose pre-Zn HS CRP values were above the median, one can see that the post-Zn values trended lower (Figure 3C). Using a paired Wilcoxon signed rank test for comparison between pre and post, the median HS CRP decreased significantly from 4.0 (IQR 2.2–4.8) to 2.3(IQR 1.4–3.5) (*p* = 0.047). Of those test subjects whose pre-Zn HS CRP results were above the median, only one test subject showed an increased HS CRP (post-Zn), that increase being +14%.

Based upon the Pearson Correlation Coefficient, there was not a meaningful association between the magnitude of the D-Lactate leak and the magnitude of the HS CRP (0.012; *p* = 0.965) for specific individual test subjects. Test subjects with higher D-Lactate leak did not correlate to having higher HS CRP levels. However, as stated, the above-median subgroups of both the D-Lactate group and the HS-CRP group did both individually respond to Zn treatment with significantly decreased blood values.

## 3. Discussion

This study has shown the following outcomes: (1) an orally administered daily dose of 53 mg Zn is sufficient to affect human duodenal gene expression as revealed by sharply upregulated expression of specific metallothioneins in transcriptome analyses of duodenal mucosal biopsies; (2) changes in expression of individual genes related to barrier function included upregulation of key junctional proteins such as Occludin and TTetraspanin, while downregulating transcription of negative modulators of TJ barrier function such as proinflammatory mediators and signaling proteins known to induce leak; (3) overall patterns of multiple gene transcription changes—as revealed by Gene Set Enrichment Analysis (GSEA)—suggested improved TJ barrier function and a decreased proinflammatory milieu; (4) protein expression levels of at least two—and possibly four—specific TJ barrier proteins were increased as a result of Zn administration; and (5) the above indicators of TJ remodeling were accompanied by a clinical indicator of functionally improved GI barrier function, namely decreased blood level of the bacterial metabolite D-Lactate after Zn treatment. Collectively, the data suggest that orally administered Zn can reduce GI barrier leak in humans, which builds upon years of cell culture and animal model findings predicting this outcome.

Individually, the three different analyses used in this study (RNA-seq, Western immunoblot and D-Lactate as a functional permeability marker) lack the statistical power (the number of test subjects/patients) that would make the findings more compelling. However, the three sets of results viewed collectively indicate a barrier effect of zinc treatment that may result in reduced leak, providing greater support to the overall conclusion. Additional studies are however clearly warranted and needed to generate a more forceful consensus of whether zinc can achieve the effect in humans that it has shown in cell culture and animal models [22,29]. This is especially true in examining the likely effect of zinc treatment on the phosphorylation state and the subcellular localization of junctional proteins. Smaller scale pilot studies such as our own can however derive value in highlighting the need for more conclusive though costly investigations with more substantive statistical power.

The use of D-Lactate as an in situ probe of GI mucosal leak has been noted in numerous prior studies of GI permeability in a variety of gastrointestinal models ranging from humans [30] to mice [31] to pigs [32] to chickens [33]. As is true for other markers of GI paracellular permeability such as lactulose/mannitol, D-Lactate is imperfect as a probe. Whereas the urinary markers of intestinal permeability can be skewed by, e.g., renal clearance issues, serum D-Lactate can be affected by indirect effects of a treatment or condition on the microbiome that produces it [25]. In addition, along with the RNA-seq and tight junctional protein data presented herein, the D-Lactate data suffers from a relatively low patient pool (*n* = 10), but all three studies derive more substance collectively when viewed in concert, with all three studies (*n* = 31) indicating zinc-induced duodenal tight junction modification and reduced leak.

A wide range of GI morbidities ranging from cancer and IBD to microbial infectious diseases result in *increased* GI barrier leak, typically involving TJ leak specifically [2]. This appears to be true of disease generally, no matter the specific epithelial tissue in question [1,34,35,36,37]. To “push the pendulum” in the other direction, that is, to induce *tightening* of the TJ and tightening of the barrier made leaky by disease, is however a difficult accomplishment given the extreme structural complexity of the TJ, not to mention the interplay of factors which can influence other elements of barrier function as well [38]. Not only is it biologically difficult to induce junctional tightening due to TJ complexity, but it is also more difficult to document mathematically (leak *decreasing*) compared to documenting the *increased* junctional leak in disease states. This current study, by focusing on a healthy, control intestinal barrier made *less* leaky by zinc treatment, faced difficulty by taking an already small number (control level leak) and reducing it further. This is very likely why the D-Lactate data of Figure 3 became statistically meaningful only when we examined the 50% of test subjects manifesting (pre-treatment) D-Lactate leak greater than the median. It is worth noting that the phenomenon of reducing leak in a control “healthy” intestinal barrier has been documented several times in human intestinal cell culture models, and for several micronutrients in addition to zinc [29,39,40]. The lower intrinsic variability of cell culture models and their data results in an easier task to prove reduced leak, than is the case for a high variability model like a patient-based study.

As opposed to classic drugs, micronutrients are often clinically disregarded by a pattern of thinking that questions their efficacy in actual clinical use. However, that thinking often derives from the observed actions of these molecules *at dietary or RDA levels*. Micronutrients can act very differently when they are presented at dosages 5-10X normal dietary levels, where they function very much like drugs, turning on signaling pathways that are normally quiescent at lower (dietary) micronutrient concentrations [17]. The action of Zn on Protein Kinase C isoforms is an excellent example [38]. The fact that humans “evolved” with these micronutrients in their diets—often at levels higher than in our modern diet—may have allowed for the evolved development of salutary effects such as barrier tightening, while also minimizing undesired side effects—the essence of natural selection [17]. The attention now paid to Vitamin D in reducing fluid leak across airway epithelial barriers in COVID (“lung water”) may be a noteworthy recent example [41]. Given the wide array of seemingly unrelated diseases—including cognitive diseases—that may trace their etiology in part to GI barrier leak [42,43,44,45,46,47], finding a micronutrient that could reduce this specific barrier leak could be clinically quite salutary and far reaching. Consider too that the operative concept here is to reduce—not eliminate—barrier leak. If a micronutrient such as Zn is only partially effective concerning this leak, it still deserves our attention. Again, using COVID as an example, ask oneself if Vitamin D achieved only a 20% reduction in respiratory morbidity, would that have been worthwhile at the height of that pandemic? If that 20% reduction in morbidity translated to a 20% decreased ICU census—or to a 20% lower need for ventilators at the height of the SARS-CoV-2 epidemic—what would that have been worth on the level of a mass population in the middle of a pandemic?

Zn has a very prodigious published literature base concerning its ability to induce remodeling of TJs and improve barrier function in cell culture and animal models. The GI epithelial barrier figures prominently in this literature, which is not surprising given Zn’s utility as a clinical treatment in diarrhea going back many centuries [45]. Numerous review articles have been written on Zn and GI barrier function. Hering (2009) and Amasheh et al. (2009) discuss Zn’s barrier actions in the context of salutary micronutrient actions generally [19,46]. Zhang (2009) discuss Zn’s barrier enhancing action in the context of alcoholic liver disease compromising the GI barrier [22]. Skrovanek et al. (2014) discuss this ability of Zn in the more general context of a variety of GI diseases [47].

The studies conducted on human GI epithelial cell cultures have yielded the best controlled and most significant data. Wang et al. (2013) and Valenzano et al. (2015) both report Zn efficacy in improving TER across CACO-2 cell layers at a 50–100 µM concentration range, though without an accompanying decrease in the leak of the paracellular probe,^14^C-D-mannitol [29,39]. A change in localization of the TJ proteins, Claudins-2 and -7, was also observed. Shao et al. (2017) also reported Zn-induced increase in TER across CACO-2 cell layers [48]. Buddington et al. (2021) observed Zn-induced decrease in leak of the paracellular probe, FITC-dextran, across CACO-2 cell layers [49].

Studies conducted on animal models and human tissue in vitro have also provided evidence of Zn improving GI barrier function. In piglet intestine, Zn did improve barrier function as evidenced by decreased lactulose/mannitol ratios, while simultaneously increasing Occludin and ZO-1 content [22]. Hu et al. (2013) observed a Zn-induced decrease in FITC-dextran diffusion across pig jejunum [50]. Zn-induced decrease in lactulose/mannitol ratio along with Occludin upregulation has been seen in mouse and rat intestine [51,52].

Whereas the above in vitro examples are of Zn inducing barrier improvement of normal GI cell layers and tissue, Zn also has shown efficacy in countering the barrier leak induced chemically or by pathogens. This has been shown for CACO-2 and T_84_ human intestinal epithelial cell culture models challenged by Ochratoxin A, enteropathogenic *E. coli*, Cryptosporidium, Shigella and Salmonella [48,53,54,55,56]. In animal tissue, acetic acid- and dinitro-benzene-sulfonic acid-induced GI leak was likewise countered by Zn treatment [57].

Our study reported here addresses whether orally administered Zn could improve (normal) GI barrier function in healthy humans in situ. Although the above cell culture and animal model studies certainly suggest that Zn would be successful in humans, the unique complexities of the human model would leave uncertainty. There are obfuscating effects of varied diets, medications, and genetic backgrounds intrinsic to any study on humans. Camilleri in 2021, citing the scarcity of actual human in vivo data on this topic, considered this issue of Zn efficacy (and that of other micronutrients) in the human GI tract to be quite undecided [58]. However, Davison et al. (2016) did show in humans that Zn partially counters the increased GI permeability brought on by heavy exercise [59]. Roy et al. (1992) and Alam et al. (1994) showed Zn-induced reduction in barrier leak in patients who were compromised by microbial-generated diarrhea [60,61]. Finally, there is the hallmark paper by Sturniolo et al. (2001) showing reduced lactulose/mannitol ratios after Zn treatment of Crohn’s Disease patients [23]. So, whereas the issue of Zn and GI permeability in humans is still marked by a scarcity of reports, those that do exist, do so in the context of Zn opposing some pathophysiological condition inducing leak. Our current study on the other hand looks at Zn improvement of GI barrier leak in healthy controls, a factor that would be essential for Zn being potentially efficacious in a prophylactic/preventative role.

A final consideration concerns the safety of the use of the level of zinc administered in the studies reported here, 53–60 mg/adult/day. Supplementation of as much as 150 mg elemental zinc per day—almost 3X the dose we were providing in this study—was observed not to lower plasma copper levels in 47 healthy volunteers, perhaps the side effect of greatest concern in terms of possible zinc toxicity. Miscellaneous minor GI side effects (metallic taste, etc.) were however observed (Samman and Roberts, 1987) [62]. Another clinical study using 150 mg zinc supplementation occurred without any GI symptoms although a 10% decrease in plasma copper was observed (but not with a 50 mg or 100 mg zinc supplementation, dosages where there was no significant effect on copper levels) (Field et al., 1987) [63]. One should also note that two commercially marketed (United States) multivitamins indicated for prevention of macular degeneration, Occuvite and Preser AREDS 2, utilize a daily zinc dose of 40 and 80 mg, respectively, and have been commercially available and in use for many years. The lack of reported untoward clinical effects of these supplements leads one to further believe that a daily dosage less than 100 mg/adult/day is likely to be safe. More research on the issue of zinc safety in a prophylactic setting is however clearly needed, especially the use of a daily regimen of zinc (in the 50–60 mg range) for an extended period.

To summarize, we attempted in these studies to demonstrate that orally administered Zn (in the 50–60 mg/adult/day range) can successfully induce TJ remodeling in human small intestine resulting in a less leaky GI barrier. More studies are required though to not only substantiate this finding but also to determine whether this orally administered Zn is active specifically from within the GI lumen (as opposed to acting from the bloodstream). There is also the question of whether similar activity can be seen in the large bowel and caecum, and whether Zn effects on the GI microbiome are involved here. A salutary effect by this safe, inexpensive ubiquitous micronutrient on something as clinically fundamental as GI barrier leak can potentially have far-ranging medical application and warrants additional research.

## 4. Methods

### 4.1. Patient Demographics and Enrollment

(1) Patient Recruitment for mRNA and Western Immunoblot Studies

All enrolled subjects provided written informed consent, and the study was approved through the Lankenau Medical Center Institutional Review Board (E-18-3817). Patients presenting for routine upper endoscopy surveillance (Barrett’s esophagus, GERD, gastritis but no history of or active small bowel disease) were contacted for possible recruitment 6–8 weeks prior to their scheduled procedure. Prior medical history exclusions were Inflammatory Bowel Disease, Irritable Bowel Syndrome, prior GI surgery, history of gastric or duodenal ulceration, history of H. pylori infection, Celiac Disease, or insulin-dependent diabetes. Patients were also excluded if they were taking a proton pump inhibitor (PPI) as these drugs can inhibit Zn uptake by the small bowel [64]. Patients on an aspirin regimen or taking any NSAID for analgesic purposes were excluded since these can aberrantly increase GI permeability. After a medication history to exclude those taking medications that would be inhibited (or their uptake affected) by Zn (amiloride-class diuretics, fluoroquinolone antibiotics, anticoagulants [except for aspirin], hormone-replacement therapy, and cholestyramine), informed written consent was obtained. Patients 18–80 years of age were recruited from any gender or ethnicity.

(2) Test Subject Recruitment for Zinc Leaky Gut Study

As above, healthy test subjects of 18–80 years of age with no history of (or active) gastrointestinal disease were enrolled and recruited from any gender or ethnicity. The same medical and medication exclusions used above were observed in this functional study. All enrolled subjects provided written informed consent, and the study was separately approved through the Main Line Hospitals Institutional Review Board (E-21-5153). Test subjects were not allowed to begin the study if a current GI infection was suspected and were withdrawn from the study if a suspected GI infection occurred during the study.

### 4.2. Zinc Administration

(1) mRNA and Western Immunoblot Study

Test subjects were instructed to take two 13.3 mg (Zn) Zn gluconate lozenges twice a day (53 mg Zn daily dose) for 14 days prior to their procedure, the final dose being taken the evening prior to the procedure. Patients were instructed to avoid foods containing phytates (seeds, nuts, etc.) as well as citrus 2 h before and 1 h after taking the Zn gluconate to avoid inhibition of Zn uptake. A matched set of patients were administered a chemically identical (sodium gluconate, molar equivalent) placebo. Patients were blinded as to which medication they were taking until after their procedure. No Zn-related adverse events were observed.

Normal (dietary) Zn intake is considered in the range of 5–10 mg/adult/day, and toxic limits are generally viewed to be above 150 mg/adult/day. Lower doses (<100 mg/adult/day) have generally been considered safe and without effect on systemic copper levels [65,66]. One hundred milligrams of Zn/adult/day had no significant effect on plasma copper levels over 3 months in an elderly population. Zn levels more than 150 mg/adult/day were required to cause changes in copper status, immune function, and HDL levels [67]. The transit time/turnover time of epithelial mucosa for normal stratified squamous esophagus is 7.5 days, whereas intestine is only 3 days [68]. Our 14-day treatment time allows for several refoliations of duodenal tissue in the presence of Zn. Therefore, we are allowing for not only Zn effects on existing, differentiated epithelia, but also Zn effects on undifferentiated epithelia (and stem cells) in the crypt regions of the duodenum.

(2) Zinc Leaky Gut Study

Test subjects were instructed to take one 30 mg (Zn) Zn gluconate tablet twice a day (daily dose of 60 mg) for 7 days prior to their procedure. The final dose was to be taken the evening prior to the procedure. Patients were instructed to avoid foods containing phytates (seeds, nuts, etc.) as well as citrus 2 h before and 1 h after taking the Zn gluconate in order to avoid inhibition of Zn uptake. A matched set of patients were administered a chemically identical (sodium gluconate) placebo. Patients were randomly assigned to the Zn or placebo subgroups by the sealed envelope method. Patients were blinded as to which medication they were taking until after they completed the entire protocol. No Zn-related adverse events occurred during the study.

### 4.3. Duodenal Biopsy Collection and Processing

During the upper endoscopy (after 14 days of Zn or placebo medication), four standard (1 mm^3^) biopsies were taken from the bulb portion of the duodenum and pooled for Western immunoblot analyses. Two biopsies were taken and pooled for mRNA analyses. Biopsies were flash frozen on dry ice in the endoscopy procedures room and stored at −196 °C (liquid nitrogen) until processed for molecular analyses.

### 4.4. RNA-seq Data Analysis

For RNA-seq analyses, RNA was extracted by homogenizing the tissue in TRI Reagent (MilliporeSigma, St. Louis, MO, USA) followed by RNA extraction using the Direct-zol RNA Mini kit (Zymo Research, Irvine, CA, USA) with an in-column DNAse treatment. Total RNA was used to generate indexed libraries using QuantSeq 3′ mRNA-seq FWD library preparation kit (Lexogen GmbH, Vienna, Austria) following manufacturer’s instructions. Final libraries were subjected to quality check on the Tape Station (Agilent, Santa Clara, CA, USA) and quantified using KAPA Library Quant Kit (for Illumina) (KAPA Biosystems, Wilmington, MA, USA). The libraries were pooled in equimolar concentration and sequenced on Nextseq 500 (Illumina, San Diego, CA, USA) using high output sequencing kit v2 generating 75 bp single reads. Data was aligned using STAR [69] algorithm against hg19 human genome version. RSEM v1.2.12 software [70] was used to estimate read counts and FPKM values using gene information from Ensemble transcriptome version GRCh37.p12. Raw counts were used to estimate significance of differential expression difference between two experimental groups using DESeq2 [71]. Gene Set Enrichment Analysis was performed using GSEA [72] estimated by the DESeq2 gene significance for gene ranking. Results that pass the *p* value (0.05) threshold were considered significant.

### 4.5. Western Immunoblot Analyses

Biopsy tissue was thawed and resuspended in 4 °C lysis buffer containing sodium dodecyl sulfate with protease and phosphatase inhibitors, and then homogenized by hand using glass mortars/pestles (Wheaton, Millville, NJ, USA), thereby generating a total cell lysate. Suspensions were then sonicated, extracted for 75–90 min at 4 °C on a rotator, and ultracentrifuged. Cellular proteins were separated on Novex tris-glycine minigels. Primary antibodies to the TJ proteins, Tricellulin, and claudins -3, -4, -5 and -7 were products of Thermo Fisher (Invitrogen) (Waltham, MA, USA). Anti-Claudin-1 was a mouse monoclonal antibody (Thermo Fisher 37-4900). Anti-Claudin-3 was a rabbit polyclonal antibody (Thermo Fisher 32-9400). Anti-Claudin-5 was a mouse monoclonal antibody (Thermo Fisher 35-25-00). Anti-Claudin-7 was a rabbit polyclonal antibody (Thermo Fisher 34-9100). Anti Tricellulin was a rabbit polyclonal antibody (Thermo Fisher 48-8400). Antisera to claudin-2 was a product of Abcam (Cambridge, United Kingdom). Antisera to Protein Kinase C-α was a product of Santa Cruz Biotechnology (Dallas, TX, USA). Secondary antibodies were purchased from Southern Biotech (Birmingham, AL, USA). Quantification of Western blot results was performed by densitometry of specific protein bands using a BioRad Chemidoc MP Imaging System (Hercules, CA, USA). Densitometry conducted on Memcode stains of total protein served as loading controls for all Western blot data [73]. Densitometry was normalized (across the entire range of patients studied) by standardizing the densitometry based on the inclusion of the first placebo-treated and patient and the first zinc-treated patient in all gels/immunoblots.

### 4.6. Blood Sampling

Within three days before beginning their Zn regimen, the test subject contributed two 5cc whole blood samples. This was repeated at the end of their 7-day Zn regimen, specifically the morning after their final Zn dose, thereby generating “pre-Zn” and “post-Zn” samples from the same individual. Blood samples were drawn by a phlebotomist in the Lankenau Medical Center outpatient testing lab. The samples were drawn in standard (red top) serum tubes. One tube was then sent for measurement of Hemoglobin A1C and CRP levels (Quest Diagnostics). The other was transported by a member of the research team to the research lab to centrifuge out blood cells and harvest serum for storage at −20 °C until testing for D-Lactate.

### 4.7. D-Lactate Analyses

D-Lactate was assayed in serum samples by a fluorometric assay kit (EnzyFluo^TM^ [BioAssay Systems, Inc.] (Hayward, CA, USA). Fluorescence was determined by a Cytation3 imaging reader (BioTek, Inc., Wisnooski, VT, USA) against a range of D-Lactate standards. Serum samples were first purified by passing through a 10k molecular weight spin column (Pierce^TM^ [Thermo Fisher, Inc., Waltham, MA, USA), then the filtrate was diluted 1:2 with glass distilled water before being assayed. An L-Lactate standard (1 mM) was used to confirm specificity of the assay for the D-stereoisomer.

### 4.8. Statistical Analyses

Where comparisons could be made between the means of placebo and zinc-treated groups (RNA-seq and Western immunoblot analyses), a two-sided Student’s t test was used. Statistical significance was claimed with a two-sided *p* value < 0.05. In comparisons of pre-treatment vs. post-treatment conditions (D-Lactate blood measurements), a paired Wilcoxon signed rank test was used.

## Figures and Tables

**Table 1:** Patient and Test Subject Demographics Relating to mRNA Microarray, Western Immunoblot and Serum D-Lactate Analyses.

RNA Microarray			
	**Mean Age (range 64–79)**	**Gender Distribution**	**Racial Composition**
Placebo Group (*n* = 5)	71.6 (range 64–79)	2 males 3 females	Caucasian
Zn-Treated Group *(n* = 6)	71.0 (range 61–76)	1 male 5 females	Caucasian (5) African American (1)
**Western Immunoblot Study**			
	**Mean Age (range 64–79)**	**Gender Distribution**	**Racial Composition**
Placebo Group (*n* = 12)	68.0 (range 55–78)	7 males 5 females	Caucasian
Zn-Treated Group *(n* = 11)	68.0 (range 54–75)	5 males 6 females	Caucasian (10) African American (1)
**Serum D-Lactate Study**			
	**Mean Age (range 64–79)**	**Gender Distribution**	**Racial Composition**
(*n* = 19)	44.0 (range 21–71)	11 males 8 females	Caucasian (16) African American (2) Asian (1)

## Data Availability

All data generated in this study can be obtained by contacting E.A. Del Rio or J.M. Mullin at the email addresses listed.

## Supplementary Materials

The following supporting information can be downloaded at: https://www.mdpi.com/article/10.3390/ijms26178540/s1.

## Abbreviations

CRP	C-Reactive Protein
GERD	Gastroesophageal Reflux Disease
GI	Gastrointestinal
GSEA	Gene Set Enrichment Analysis
HS CRP	High Sensitivity C-Reactive Protein
IBD	Inflammatory Bowel Disease
LG	Leaky Gut
NSAID	Non-Steroidal Anti-Inflammatory Drug
RDA	Recommended Daily Allowance
TER	Transepithelial Electrical Resistance
TJ	Tight Junction
Zn	Zinc
