# Supplementary material for: Orally Administered Zinc Gluconate Induces Tight Junctional Remodeling and Reduces Passive Transmucosal Permeability Across Human Intestine in a Patient-Based Study
Source: Int J Mol Sci. 2025 Sep 2;26(17):8540. doi: 10.3390/ijms26178540 (PMC12429388; doi:10.3390/ijms26178540)
Supplement: Supplementary file 1 [file ijms-26-08540-s001.zip › ijms-3720966-supplementary.pdf]

Figure S1, Supplemental Data

Claudin-2 Duodenal Whole Cell Lysates: Zinc Study

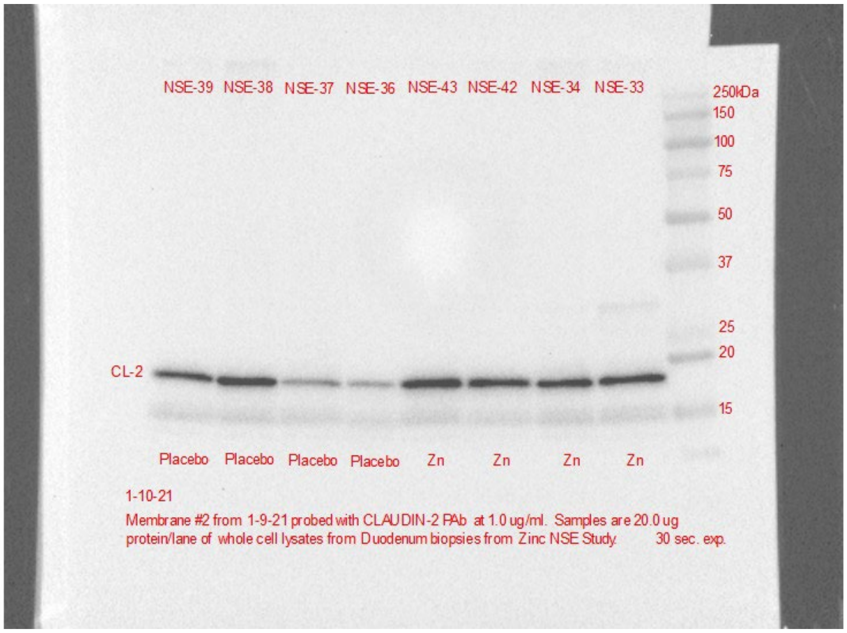

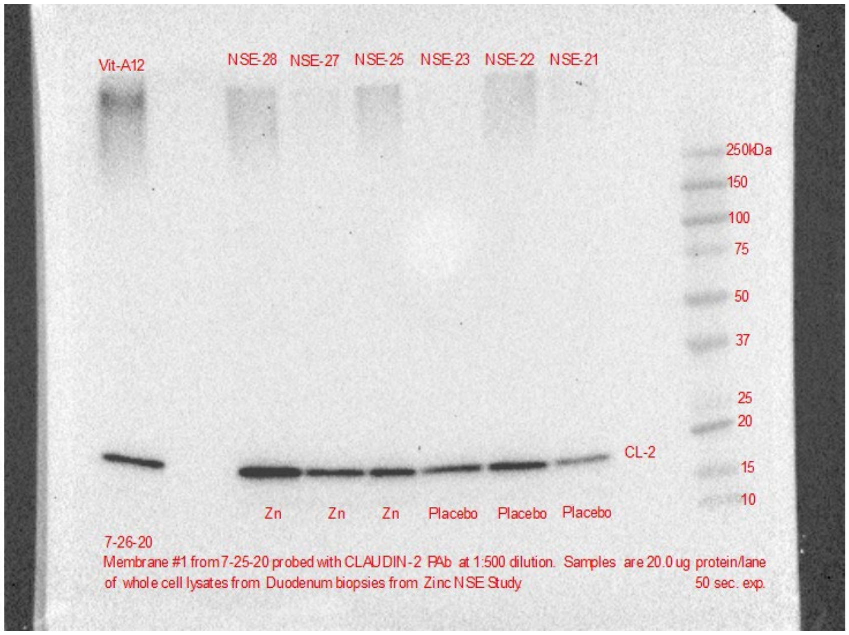

Claudin-2 Duodenal Whole Cell Lysates: Zinc Study (continued)

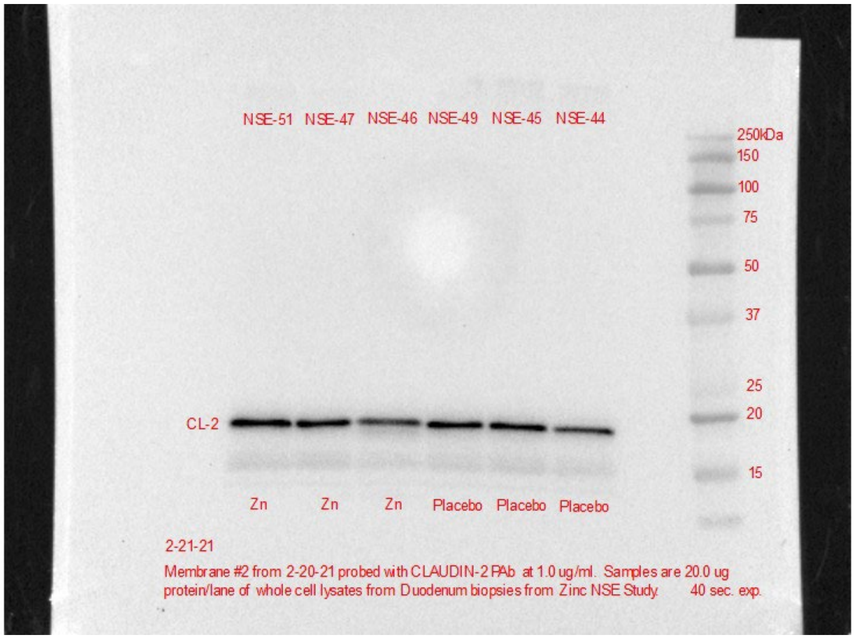

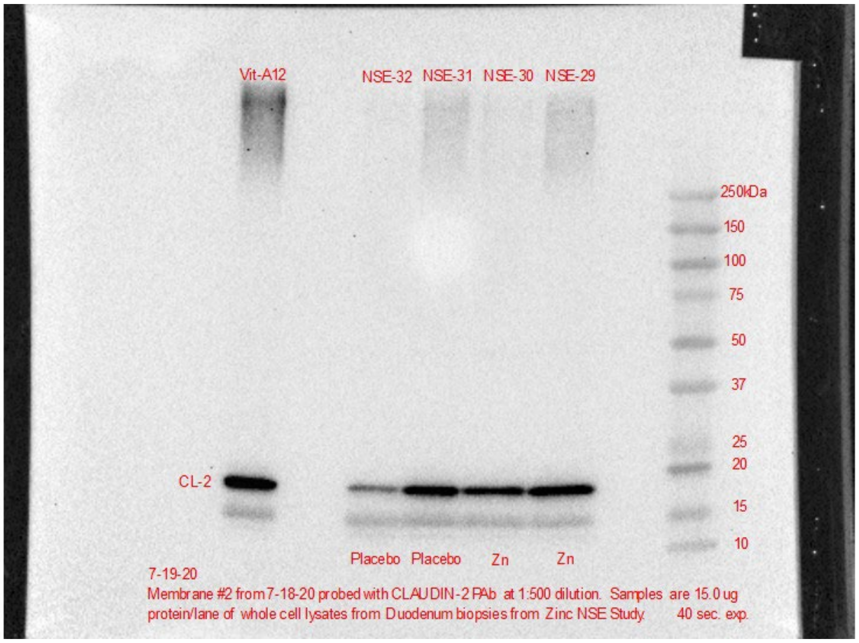

Figure S1 Supplemental. Claudin-2 Western Immunoblot Raw Data. Duodenal biopsy whole cell lysates for 11 zinc-treated patients (patient NSE-46 was excluded due to abnormal Memcode protein banding) and 12 placebo-treated patients are shown. Individual patients are identified as code numbers, NSE-##. Quantitation of densitometry of Tricellulin bands in the summary figure (manuscript, Figure 2) required normalization among the 4 different blots shown above, as there were too many patient samples to run in a single gel.

Figure S2, Supplemental Data

Tricellulin Duodenal Whole Cell Lysates: Zinc Study

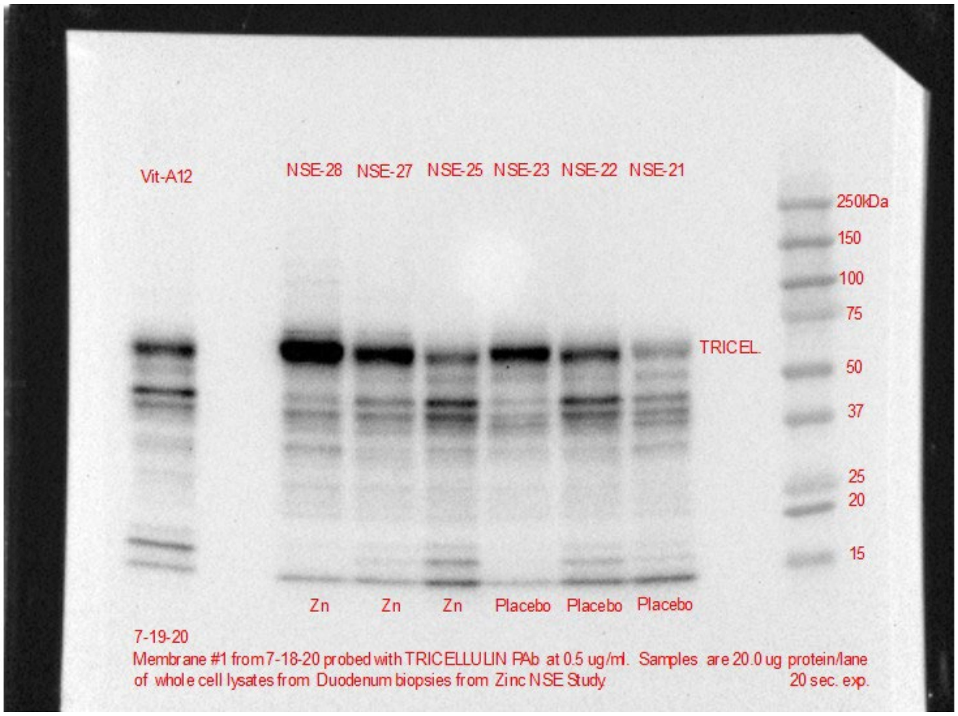

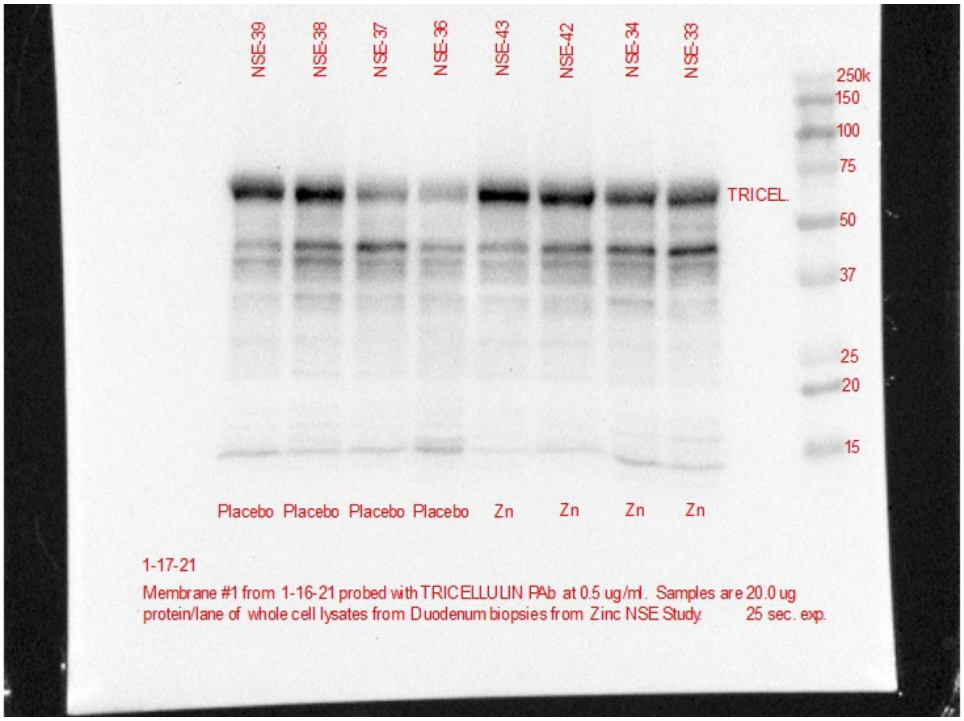

Tricellulin Duodenal Whole Cell Lysates: Zinc Study (continued)

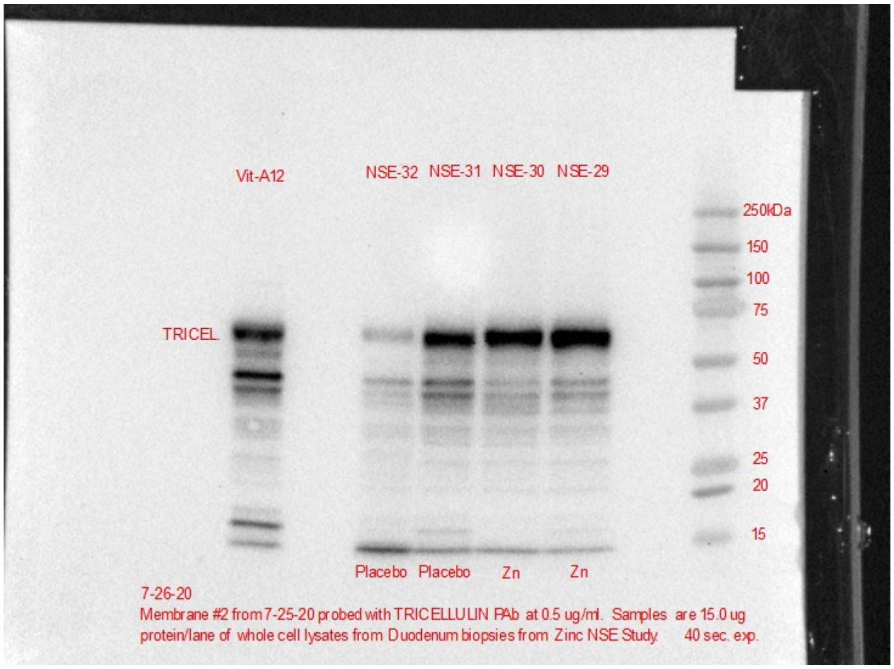

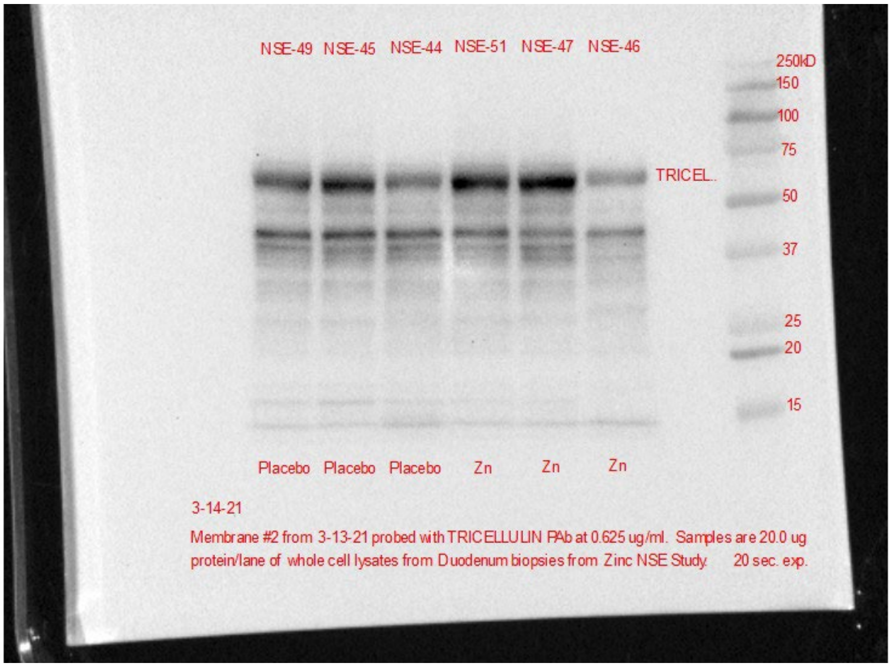

Figure S2 Supplemental. Tricellulin Western Immunoblot Raw Data. Duodenal biopsy whole cell lysates for 11 zinc-treated patients (patient NSE-46 was excluded due to abnormal Memcode protein banding) and 12 placebo-treated patients are shown. Individual patients are identified as code numbers, NSE-##. Quantitation of densitometry of Tricellulin bands in the summary figure (manuscript, Figure 2) required normalization among the 4 different blots shown above, as there were too many patient samples to run in a single gel.
